# Supplementary material for: Fast-charging high-energy lithium-ion batteries via implantation of amorphous silicon nanolayer in edge-plane activated graphite anodes
Source: Nat Commun. 2017 Oct 9;8:812. doi: 10.1038/s41467-017-00973-y (PMC5634447; doi:10.1038/s41467-017-00973-y)
Supplement: Supplementary file 1 — Supplementary Information [file 41467_2017_973_MOESM1_ESM.pdf]

Supplementary information

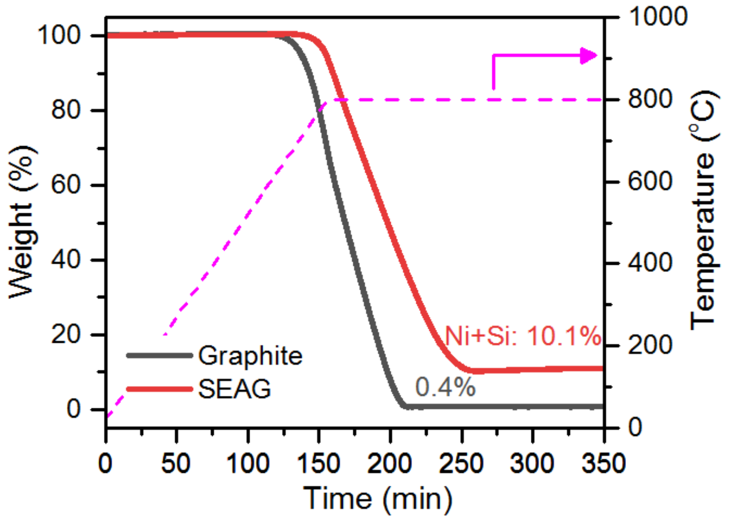

Supplementary Figure 1. Thermogravimetric analysis of pristine graphite and SEAG.

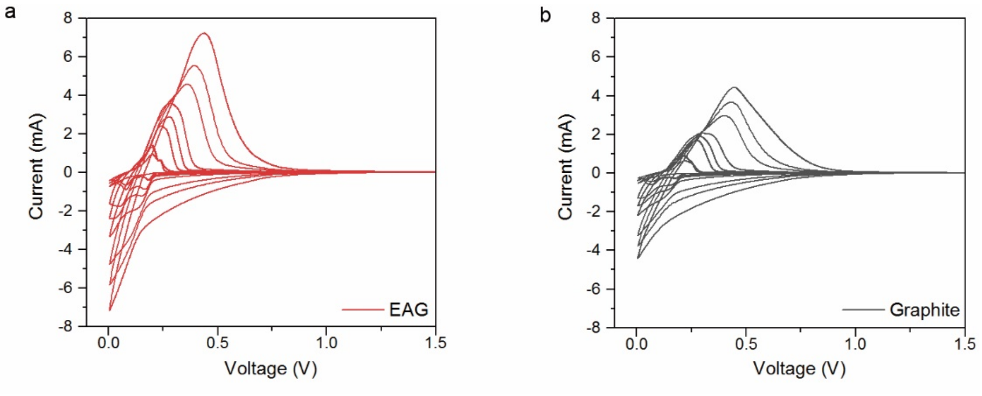

Supplementary Figure 2. Cyclic voltammograms of (a) EAG and (b) graphite.

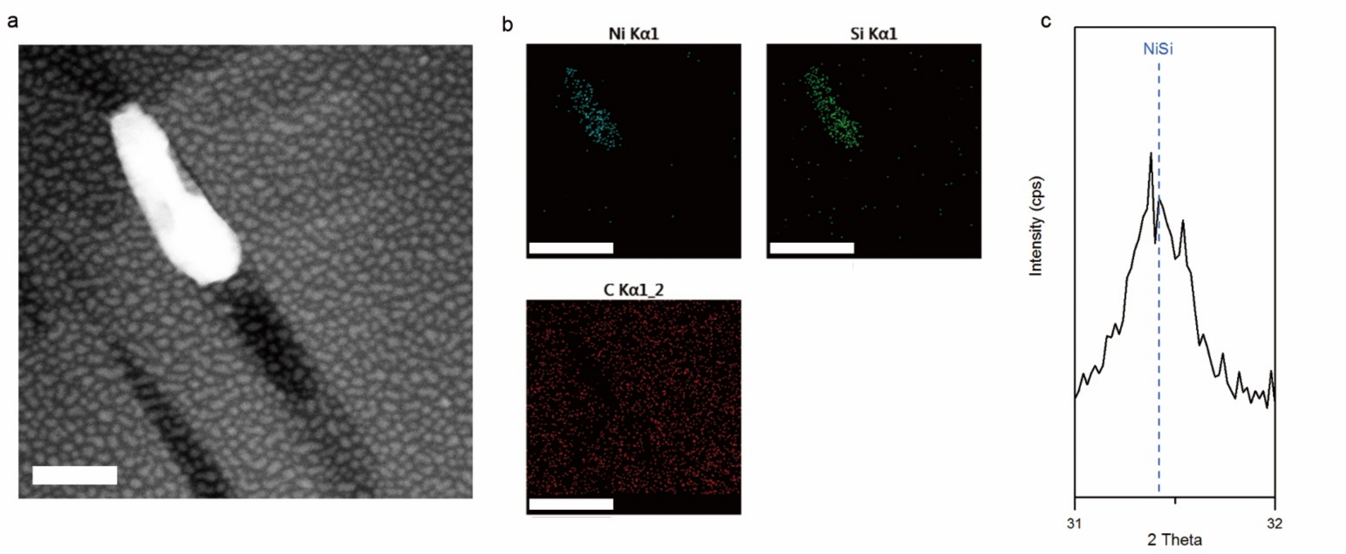

Supplementary Figure 3. Identification of nickel silicide formation under SiH_4 atmosphere below the gas decomposition temperature (≤ 400 °C). (a-b) Nickel silicide embedded in graphite. Note that there was no silicon element on the graphite, and silicon was detected only nickel silicide particle. This TEM image with EDS result clarify that the nickel silicide is earlier formed before the beginning of SiH_4 gas decomposition. (c) XRD characteristic peak of NiSi phase. Scale bars, (a) 50 nm, (b) 100 nm.

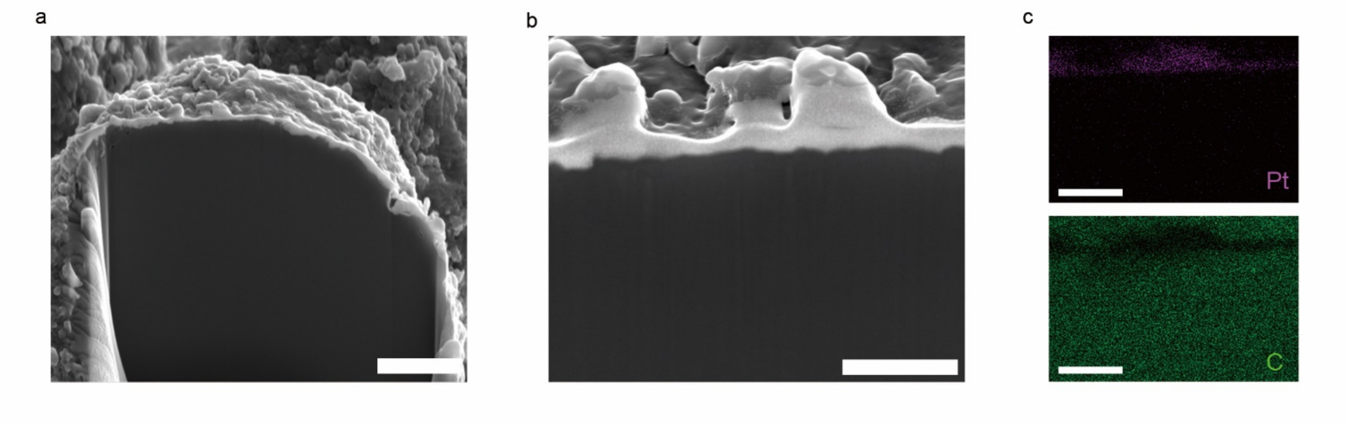

Supplementary Figure 4. (a) Cross sectional SEM image of pristine graphite with EDS mapping analysis. The pristine graphite has dense internal structure without any void. Scale bars, (a) 5 μm, (b) and (c) 1 μm.

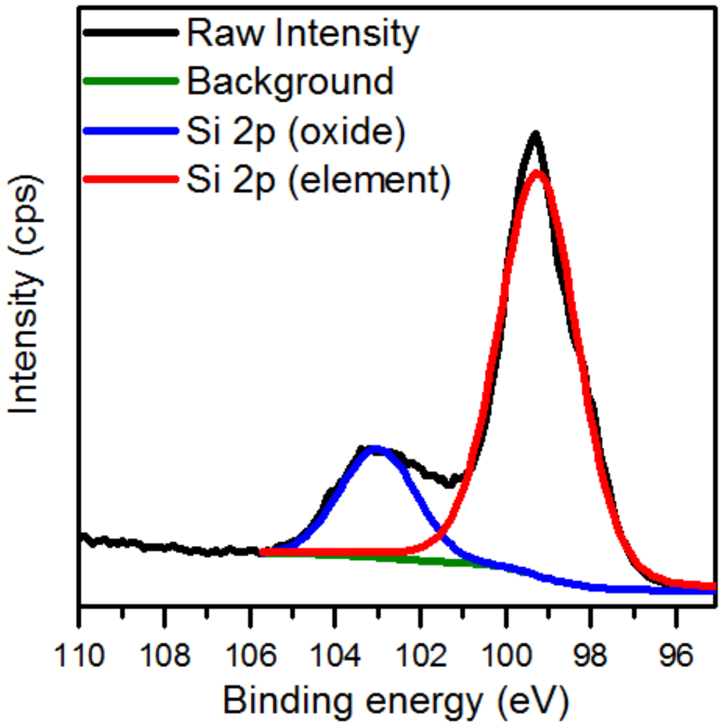

Supplementary Figure 5. X-ray photoelectron spectra of the SEAG composite. This result indicates that the native oxide layer exists on the surface and the thickness of this layer would be very thin. On the basis of the effective electron escape depth for Si 2p in elemental Si (~15 Å)[1], such native oxide layer is considered as thinner than 15 Å as otherwise the Si 2p peak cannot be detected.

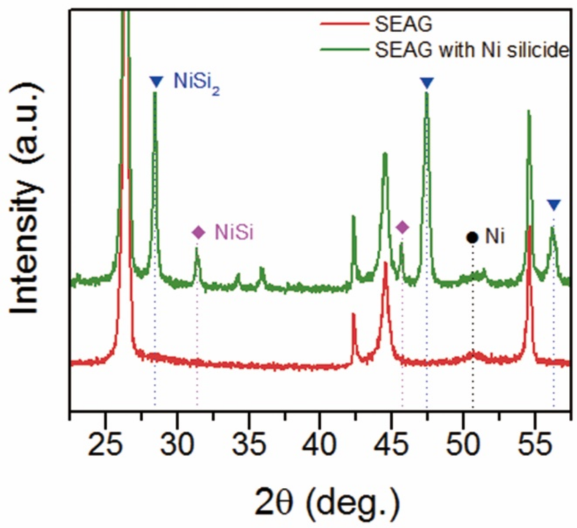

Supplementary Figure 6. Comparison of XRD analysis results between SEAG and SEAG with Ni silicide. Unlike SEAG with Ni silicide, in SEAG, there were no any characteristic peaks indicating Ni silicide phases.

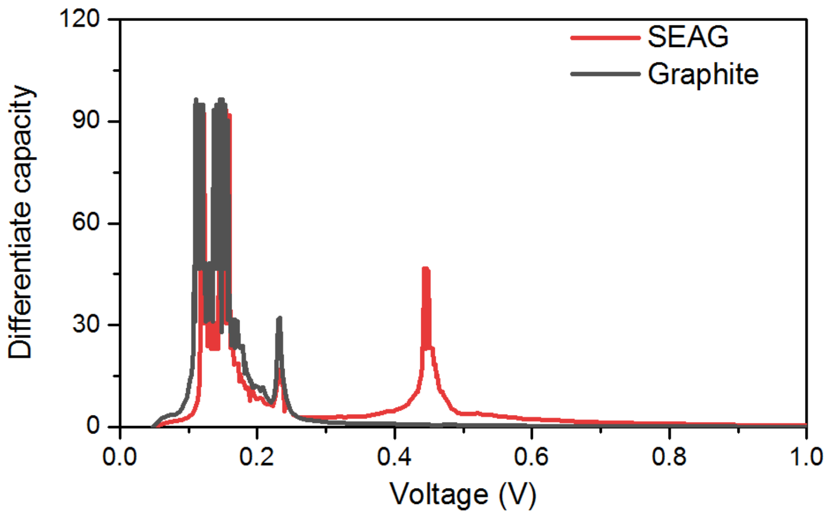

Sample	Area ratio		Capacity contribution	
SEAG	Below 0.3 V	Above 0.3 V	Below 0.3 V	Above 0.3 V
	60.9%	39.1%	319.7 mAh g^{-1}	205.3 mAh g^{-1}
Graphite	Below 0.3 V	Above 0.3 V	Below 0.3 V	Above 0.3 V
	97.1%	2.9%	313.7 mAh g^{-1}	9.3 mAh g^{-1}

Supplementary Figure 7. Differential capacity plot and capacity contribution of each sample. Unlike the graphite, there is an additional oxidation region above 0.3 V in the SEAG. In this regard, it is clear that this additional capacity of SEAG above 0.3V is mainly contributed to Si and the capacity from Si is around 205 mAh g^{-1}.

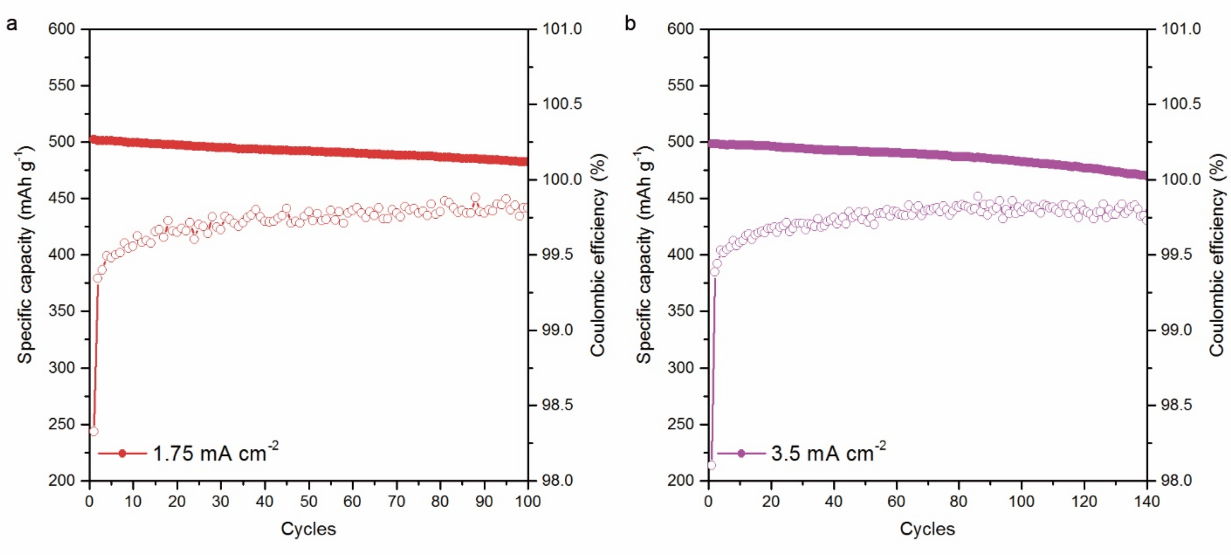

Supplementary Figure 8. Cycling performance of SEAG at various current densities. (a) Cycle life of SEAG at 1.75 mA cm^{-2}. (b) At 3.5 mA cm^{-2}. Charge and discharge current density are the same.

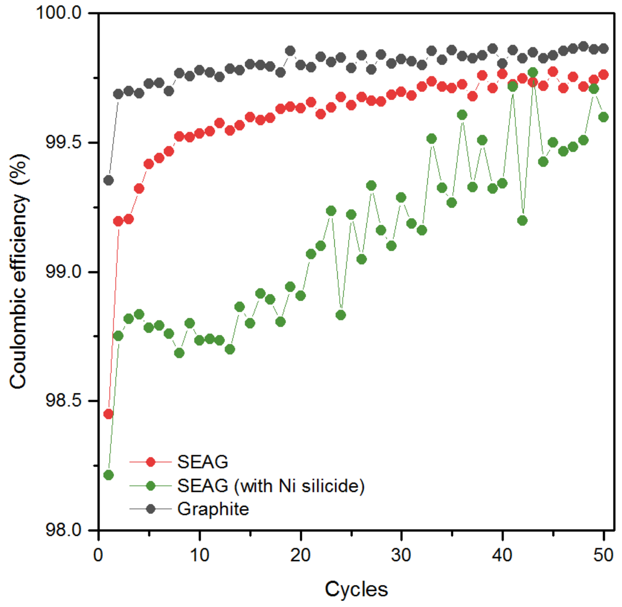

Supplementary figure 9. Magnified plot of Coulombic efficiency of each sample for cycles.

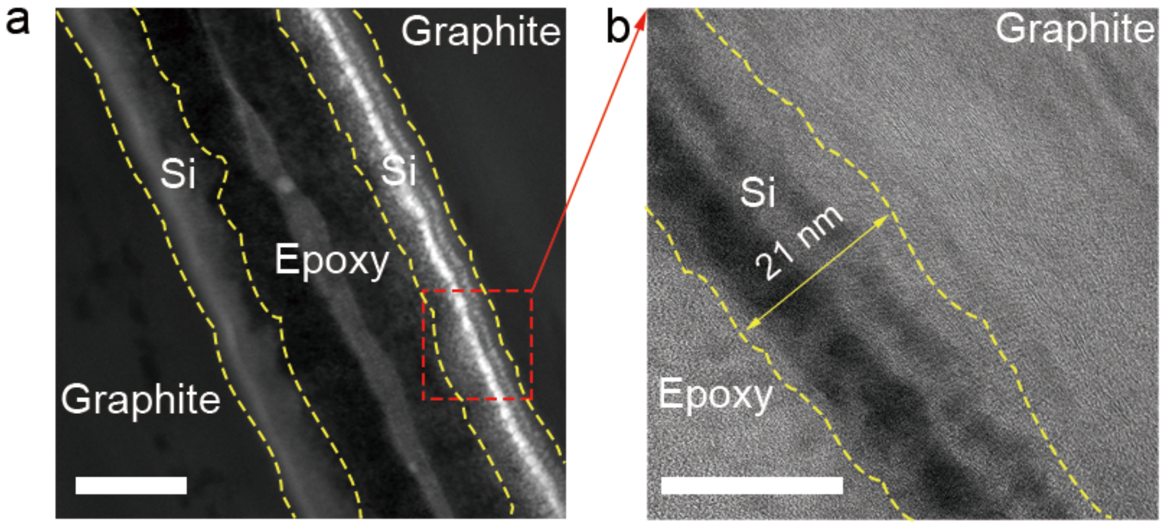

Supplementary Figure 10. TEM analysis of SEAG after 50 cycles. (a) HAADF-STEM image of silicon nanolayer on the activated hole. (b) HR-TEM image of the silicon nanolayer. The epoxy deposition on the sample was conducted as protective layer for TEM sampling. The Si nanolayer is well adhered to the graphite without any delamination or crack of the layer and the deposited Si is also retained in a layer morphology very well without any aggregation of each other even after 50 cycles. These structural stabilities of SEAG during cycles result in the excellent cycling performance. Scale bars, (a) 50 nm, (b) 20 nm.

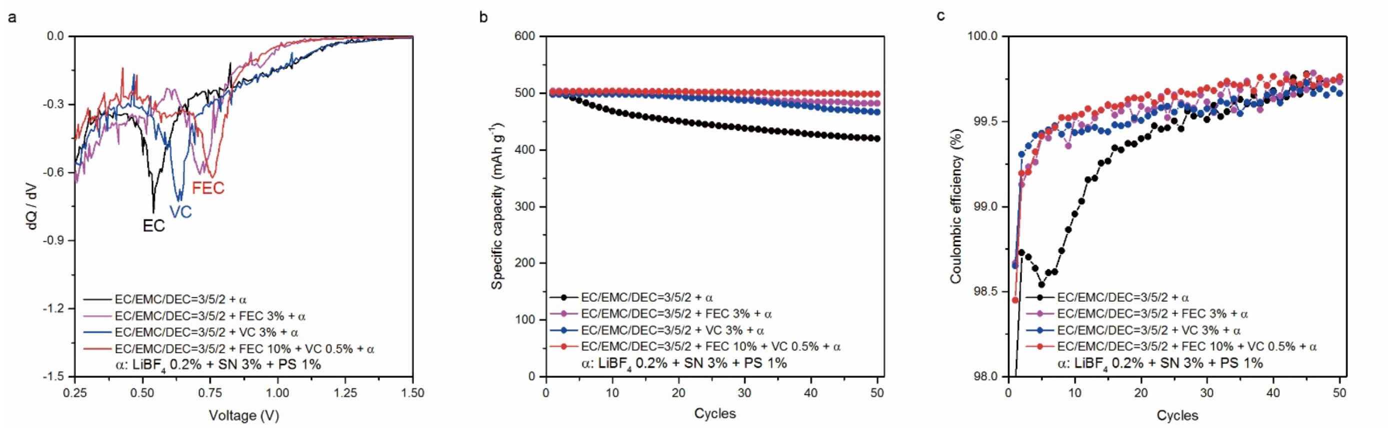

Supplementary Figure 11. Electrochemical properties of SEAG in various additive composition. (a) dQ/dV plot during lithiation process at the first cycle. (b) Cycling performance at current density of 1.75 mAh cm-2. (c) Coulombic efficiency for cycles. It is clearly shown that the presence of additives suppresses the reduction of electrolyte such as EC [2, 3]. In addition, the SEAG composite exhibited good cycling performance even in relatively low content of FEC and VC additive (3 wt%).

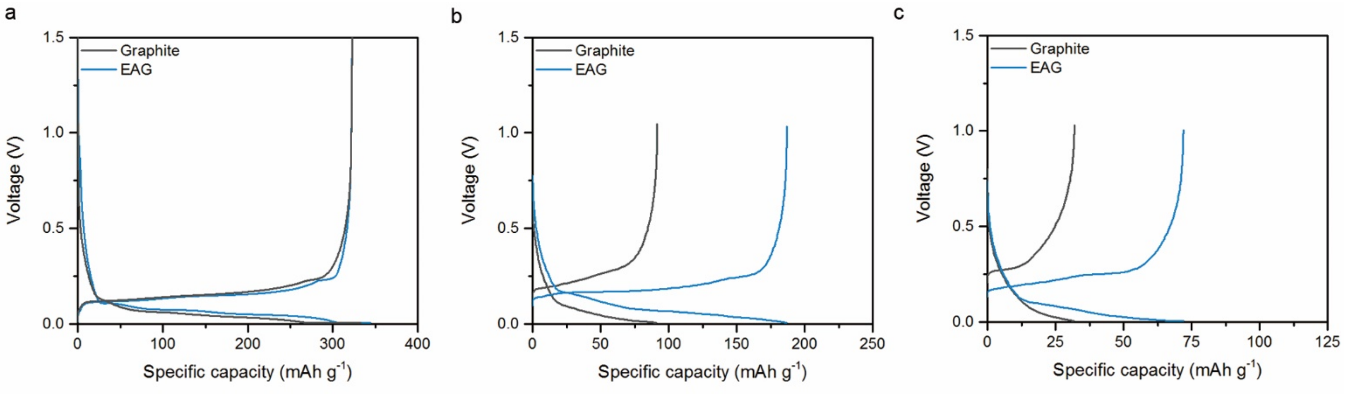

Supplementary Figure 12. Voltage profile of EAG and pristine graphite at various current densities. (a) Voltage profile in the first cycle using both galvanostatic and potentiostatic method in charging step. Only galvanostatic charge/discharge profiles at (b) 3.5 mA cm^{-2} and (c) 7.0 mA cm^{-2}. EAG exhibited higher capacities at all current densities than graphite.

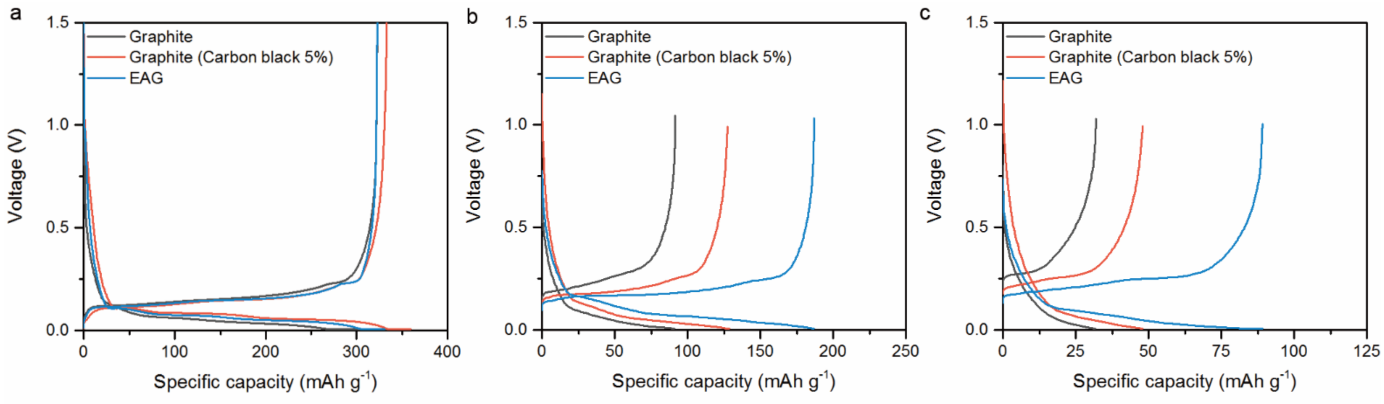

Supplementary Figure 13. Voltage profile of EAG, pristine graphite, and graphite containing 5 wt% of carbon black at various charging current densities. (a) Voltage profile in the first cycle using both galvanostatic and potentiostatic method in charging step. Only galvanostatic charge/discharge profiles at (b) 3.5 mA cm^{-2} and (c) 7.0 mA cm^{-2}.

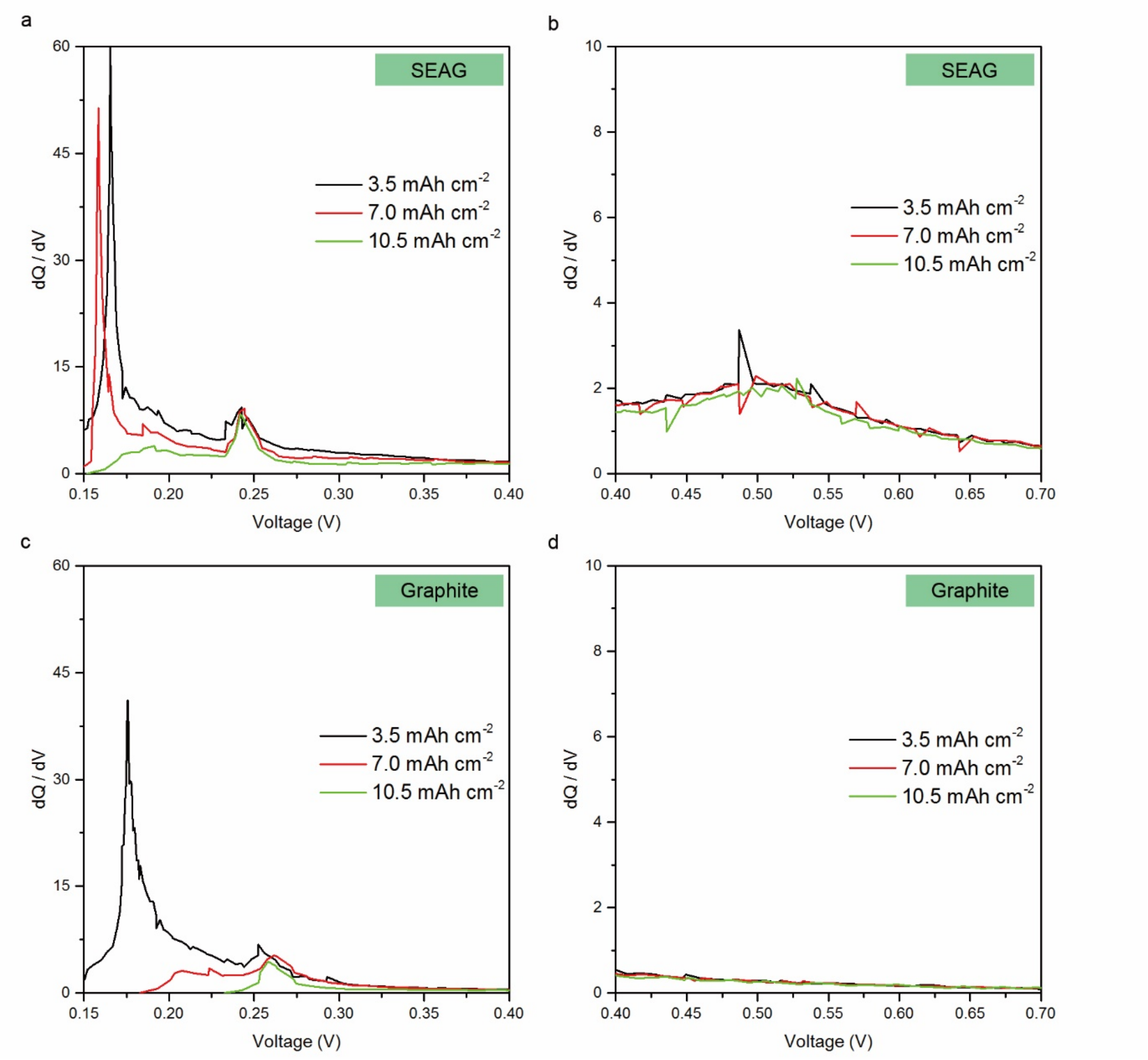

Supplementary Figure 14. dQ/dV plot for delithiation process at various charge current density. All tests were measured by only the galvanostatic method. (a) and (b) dQ/dV plot of SEAG. (c) and (d) dQ/dV plot of pristine graphite.

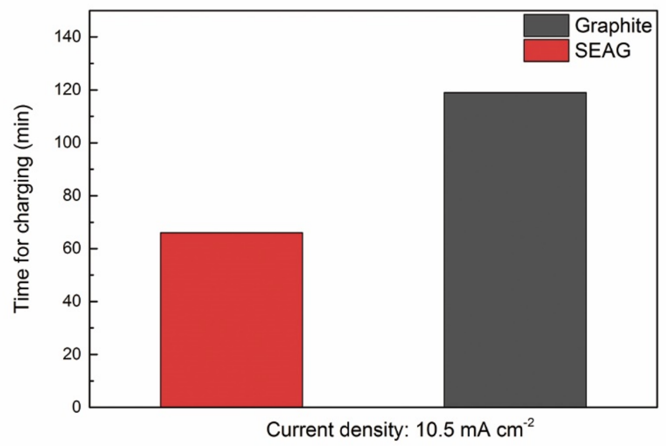

Supplementary Figure 15. The required time for fully lithiated state at 10.5 mA cm^{-2} in half-cell test.

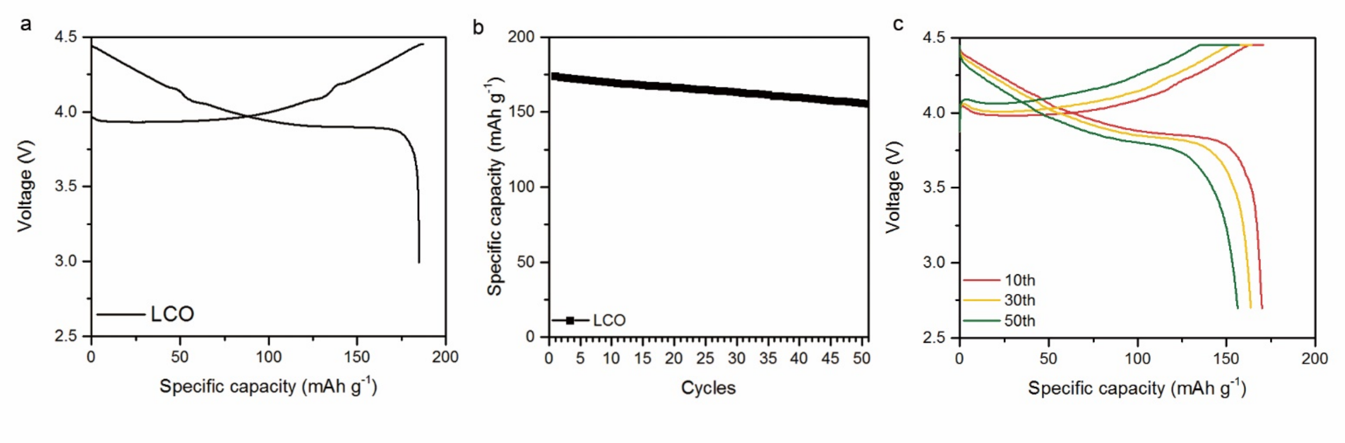

Supplementary Figure 16. Electrochemical performances of $LiCoO_2$ cathode in half-cell. (a) Voltage profile at the first cycle. (b) Cycling test at 0.5C-rate for 50 cycles. (c) Voltage profiles at each cycle. All tests were conducted in the potential window from 3.0 V to 4.45 V.

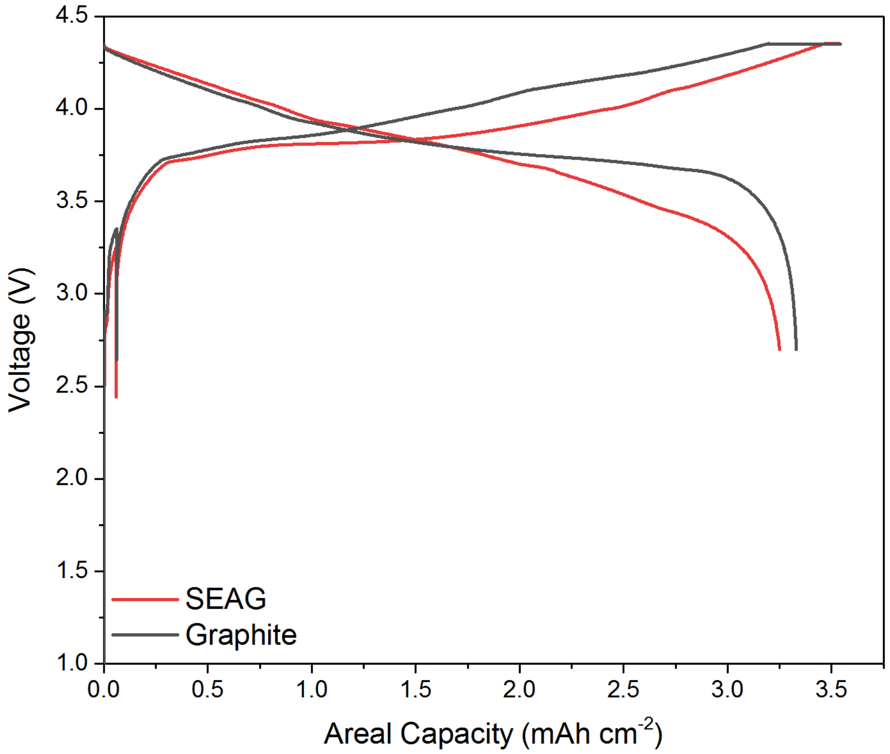

Supplementary Figure 17. Charge/discharge voltage profile of SEAG and graphite at the first cycle in full-cell configuration.

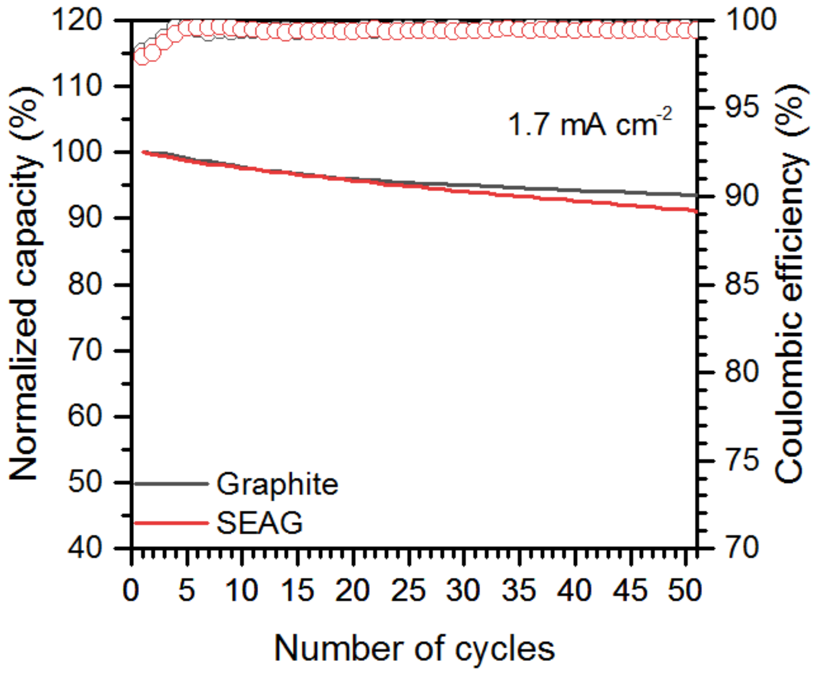

Supplementary Figure 18. Cycling performance of the full-cell with anodes of graphite and the SEAG composite under current density of 1.7 mA cm^{-2}. The cycling tests were performed in the voltage window between 2.7 and 4.35 V.

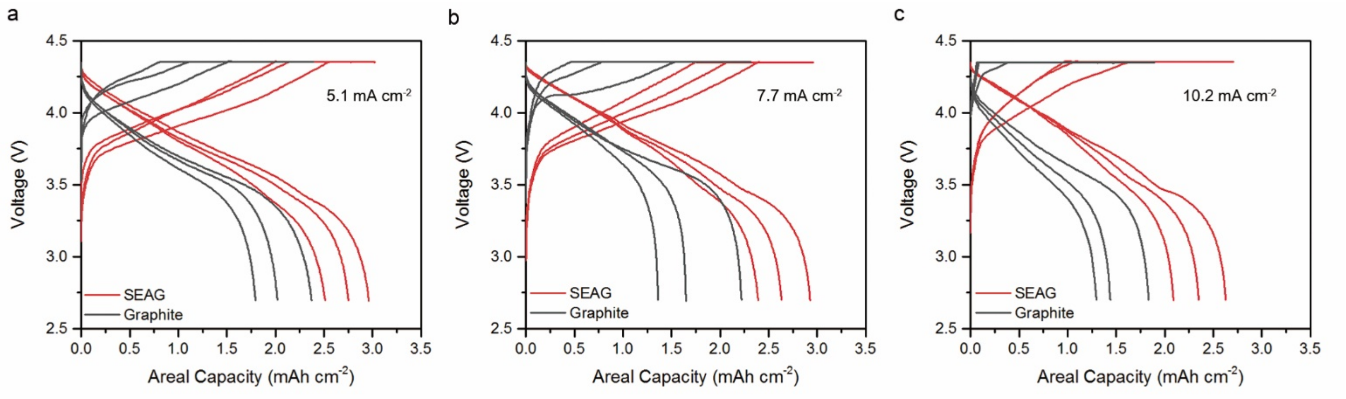

Supplementary Figure 19. Charge/discharge voltage profiles of SEAG and graphite at 10th, 30th, and 50th cycle in full-cell test. Charging current densities of (a) 5.1 mA cm^{-2}, (b) 7.7 mA cm^{-2}, and (c) 10.2 mA cm^{-2} were applied.

Sample	Element	Result	Tool
SEAG	Silicon	6.3 wt%	ICP-OES Varian 700-ES
	Nickel	2.8 wt%	
	Carbon	-	
EAG	Carbon	96.3 wt%	EA Thermo Flash 2000
Carbon deposited EAG	Carbon	96.5 wt%	

Supplementary Table 1. Quantitative analysis of each sample.

Approach	Example	CE at the first cycle (%)	Electrode composition (wt%) (AM[a] : CM[b]: BM[c])	Mass loading level (mg cm^{-2})	Areal capacity (mAh cm^{-2})	Electrode density (g cm^{-3})	Fast charge capability[d]	Ref.
**Graphite-based anodes**	KOH etched graphite	92.4	96 : 1 : 3	5	1.74	1.5	13.8%	4
	VGCF/Carbon nano-horn/ graphite composite	84.0	95 : 0 : 5	6-7	2.03-2.36	-	7%	5
	Magnetically aligned graphite flake	80.0	80 : 10 : 10	5	1.48	-	17.4%	6
	**Edge-plane activated graphite (EAG)**	**93.8**	**96 : 1 : 3**	**10.5**	**3.34**	**1.6**	**20.5%**	**This work**

a. Active material, b. Conductive agent, c. Binder material
d. Normalized galvanostatic lithiation capacity at 2C rate in half-cell

Supplementary Table 2. The fast charging performance of graphite based anodes in previous works.

**Volumetric energy density**			
$\frac{\text{(Areal cell capacity)} \times \text{(Average voltage)}}{\text{(Total thickness of cathode and anode)}}$			
**Full-cell electrode information**			
	LCO	Graphite	SEAG
Mass loading level (mg cm^{-2})	20	12.1	7.1
Electrode density (g cm^{-3})	3.6	1.6	1.6
Electrode thickness (μm) (excepting current collector)	55	75	45
**Full-cell information**			
At a charging current density of 0.34 mA cm^{-2}			
	LCO	Graphite	SEAG
Areal cell capacity (mAh cm^{-2})	-	3.33	3.25
Average voltage (V)	-	3.86	3.79
Thickness at fully-charged state (μm)	55 (fixed)	84	54
At a charging current density of 10.2 mA cm^{-2}			
	LCO	Graphite	SEAG
Areal cell capacity (mAh cm^{-2})	-	2.71	3.09
Average voltage (V)	-	3.70	3.74
Thickness at fully-charged state (μm)	55 (fixed)	84	54
**Energy density calculation**			
Applied charging current density		Graphite	SEAG
0.34 mA cm^{-2}		932 Wh L^{-1}	1126 Wh L^{-1}
10.2 mA cm^{-2}		726 Wh L^{-1}	1060 Wh L^{-1}

Supplementary Table 3. Volumetric energy density calculation and electrode information.

Supplementary Note 1

The relative electrochemical active surface area was evaluated by the relationship between scan rate and peak current from the Randles-Sevcik equation (1). According to this equation, the slope in Fig. 1d is proportional to the active surface area multiplied by the square root of the Li ion diffusivity. The slopes were 5.80 for EAG and 3.03 for pristine graphite. Since the intrinsic Li ion diffusivity of graphite anode is immutable, we can speculate that EAG has almost twice as larger electrochemically active surface area than that of pristine graphite.

$$I_p = (2.69 \times 10^5) n^{3/2} A D^{1/2} C_0 v^{1/2} \qquad (1)$$

I_p [mA] = peak current, n = the number of transferred electron,

A [cm^2] = the active surface area, D [$cm^2 \cdot s^{-1}$] = the diffusion coefficient of Li ion,

C_0 = the bulk concentration of Li ion, and v [$mV \cdot s^{-1}$] = scan rate

Supplementary Note 2

On the basis of the ICP result (Table S1), we have estimated the total capacity of SEAG composite considering the content of Ni (2.8 wt%).

$$({}^{a}323\ mAh\ g^{-1} \times \frac{90.9}{100}) + ({}^{b}3600\ mAh\ g^{-1} \times \frac{6.3}{100}) \cong 520.4\ mAh\ g^{-1}$$

a. Specific capacity of edge-activated graphite (EAG)

b. Theoretical specific capacity of silicon

According to this estimation, the capacity of SEAG in our experiment (525 mAh g^{-1}) agrees well with the calculated capacity (520.4 mAh g^{-1}).

Supplementary References

1 Himpsel, F. J., McFeely, F. R., Taleb-Ibrahimi, A., Yarmoff, J. A. & Hollinger, G. Microscopic structure of the SiO2/Si interface. *Physical Review B* **38**, 6084-6096 (1988).

2 Choi, N.-S. *et al.* Effect of fluoroethylene carbonate additive on interfacial properties of silicon thin-film electrode. *J. Power Sources* **161**, 1254-1259 (2006).

3 Dalavi, S., Guduru, P. & Lucht, B. L. Performance Enhancing Electrolyte Additives for Lithium Ion Batteries with Silicon Anodes. *J. Electrochem. Soc.* **159**, A642-A646 (2012).

4 Cheng, Q., Yuge, R., Nakahara, K., Tamura, N. & Miyamoto, S. KOH etched graphite for fast chargeable lithium-ion batteries. *J. Power Sources* **284**, 258-263 (2015).

5 Yuge, R., Tamura, N., Manako, T., Nakano, K. & Nakahara, K. High-rate charge/discharge properties of Li-ion battery using carbon-coated composites of graphites, vapor grown carbon fibers, and carbon nanohorns. *J. Power Sources* **266**, 471-474 (2014).

6 Billaud, J., Bouville, F., Magrini, T., Villevieille, C. & Studart, A. R. Magnetically aligned graphite electrodes for high-rate performance Li-ion batteries. *Nat. Energy* **1**, 16097 (2016).
